# Advancements and Updates on Operative Techniques in Spinal Deformity

**DOI:** 10.3390/jcm11216325

**Published:** 2022-10-27

**Authors:** Jessica Ryvlin, Rafael De la Garza Ramos, Mousa K. Hamad, Reza Yassari

**Affiliations:** 1 Spine Research Group, Montefiore Medical Center, Albert Einstein College of Medicine, New York, NY 10467, USA; 2 Department of Neurological Surgery, Montefiore Medical Center, Albert Einstein College of Medicine, New York, NY 10467, USA

Spinal deformity involves a spectrum of abnormal spinal curvatures deviating from normal alignment. The resultant disease is described based on its three-dimensional curvature characteristic, with scoliosis occurring in the coronal plane and kyphosis/lordosis occurring in the sagittal plane. In the United States alone, an estimated 27.5 million elderly individuals suffer from some degree of spinal deformity, most often caused by degenerative spine disease [1,2]. However, pediatric and adolescent populations experience spinal deformity as well, primarily involving idiopathic scoliosis that has a prevalence of 1–3% [3]. For select patients, the gold standard treatment to restore normal spinal alignment is multilevel surgical instrumentation with vertebral fusion [4,5]. While these operations result in notable improvements in alignment and quality of life, the risk of operative complications is significant, including infection, blood loss, or neurologic injury [6]. Recent advancements in predictive analytics and intraoperative techniques have the potential to improve the safety of and outcomes in this surgical population. 

One notable area of recent improvement is within predictive modeling, which allows surgeons to select patients that are more likely to experience surgical success, medically optimize preoperatively, and plan for postoperative disposition. Two recent studies have analyzed the ability of novel software to anticipate perioperative complications in adult spinal deformity surgery, including the need for a blood transfusion and the development of proximal junctional kyphosis (PJK). In a retrospective study conducted by De la Garza Ramos et al., a feedforward artificial neural network (ANN) was built utilizing the American College of Surgeons National Surgical Quality Improvement Program (NSQIP) database to predict the need for intra- or postoperative red blood cell transfusion [7]. Following training, validating, and testing several ANN models, one advanced learning algorithm with 18 inputted patient-specific risk factors and 2 hidden layers was observed to achieve high sensitivity (0.80), positive predictive value (0.76), overall accuracy (0.77), and discrimination (0.84), indicating the correct prediction of blood transfusion requirements in 80% of patients. While specific predisposing factors are unable to be identified using ANN models, this study highlights a novel artificial intelligence approach that could be applied to a myriad of complications following adult spinal deformity surgery. Predictive modeling has also been utilized in the form of three-dimensional imaging, as conducted by Asada et al. in an effort to predict postoperative PJK [8]. In this case–control study, preoperative dynamic spinopelvic parameters were measured using three-dimensional gait analysis, a more comprehensive approach to replace static *X*-ray imaging. After measuring 20 dynamic spinal parameters, authors found that a larger preoperative dynamic thoracic–pelvic spinal angle (T-PSA) was independently associated with PJK incidence following surgery. New imaging techniques, such as gait-exacerbated spinal alignment, have the potential to expand our understanding of spinal compensation in adult spinal deformity, which can be clinically utilized as a non-invasive method for predicting outcomes. 

Similar to new technology outside of the operating room, advancements in intraoperative techniques have been utilized to improve the success rates of spinal deformity surgery. In a study conducted by Funao et al., sacropelvic fixation with S2-alar-iliac screws (S2AI) based on patient sex was evaluated in adults, given the innate difference in pelvic size and shape between males and females [9]. Three-dimensional pelvic computed tomography was used to determine ideal trajectory pathways for S2AI screw placement, which was determined to be more laterally angled in the axial plane in females (right 47.7°, left 46.1°) than in males (right 45.3°, left 44.3°), and more horizontally angled in the coronal plane in females (right 33.7°, left 34.5°) than in males (right 36.5°, left 37.0°). Additionally, shorter distances were observed between the midline and starting points of S2AI screw placement in females than in males, as well as positive correlations between patient height and maximal lengths and minimal areas of S2AI pathways. Minimally invasive operative techniques have also experienced recent improvements, as can be observed in the study conducted by Endo et al. This study evaluated a new hybrid approach to posterior fixation (PF following lateral lumbar interbody fusion (LLIF) in adult spinal deformity [10]. Comparing traditional LLIF + PF versus LLIF + hybrid PF using percutaneous pedicle screws, the authors of this study observed similar improvements in all measured radiographic spinal parameters and in patient-reported pain-relief scores. However, the incorporation of minimally invasive PF resulted in significantly reduced operative blood loss and postoperative complications, including rod fracture, indicating that LLIF with hybrid PF may be superior to conventional techniques. Minimally invasive approaches have been adapted further to the adolescent population, with the recent development of a new minimally invasive scoliosis surgery (MISS) aimed at decreasing complications, such as wound infection and rod dislodgement observed following conventional open scoliosis surgery (COSS) [11]. In the study conducted by Park et al., the authors described a “coin-hole technique” that includes the use of tubular retractors, guide wire with cannulated instruments for pedicle screw placement, fusion bed preparation with a specially designed reamer, application of fusion materials prior to pedicle screw placement, all-pedicle screw fixation, and thoracoplasty via undermining the skin. The preliminary results show comparable postoperative patient satisfaction and spinal parameter improvements, with the exception of the Cobb angle, which was significantly lower in MISS than in COSS. As a result, MISS was deemed more appropriate for patients with Cobb angles of less than 80°. Although still in development, the use of minimally invasive approaches in the context of spinal deformity surgery is supported by a trend in decreased complications while maintaining similar improvement in the outcomes.

Spinal deformity surgery, in both adult and pediatric patients, presents with considerable risk for postoperative complications. However, these interventions are necessary for the improvement in spinal alignment and quality of life. The five studies included in this discussion feature recent advancements in patient-specific predictive modeling using artificial intelligence and advanced three-dimensional imaging, as well as novel intraoperative techniques utilizing gender-specific instrumentation and minimally invasive approaches. Ultimately, these advancements have the potential to decrease postoperative complications, increase surgical success, and improve patient satisfaction. 

## References

[1] Safaee M.M., Ames C.P., Smith J.S. (2020). Epidemiology and Socioeconomic Trends in Adult Spinal Deformity Care. Neurosurgery.

[2] Kim H.J., Yang J.H., Chang D.G., Suk S.I., Suh S.W., Song K.S., Park J.B., Cho W. (2020). Adult Spinal Deformity: Current Concepts and Decision-Making Strategies for Management. Asian Spine J..

[3] Menger R.P., Sin A.H. (2022). Adolescent and Idiopathic Scoliosis.

[4] Wiggins G.C., Shaffrey C.I., Abel M.F., Menezes A.H. (2003). Pediatric spinal deformities. Neurosurg. Focus.

[5] Good C.R., Auerbach J.D., O’Leary P.T., Schuler T.C. (2011). Adult spine deformity. Curr. Rev. Musculoskelet. Med..

[6] Kanter A.S., Shaffrey C.I., Mummaneni P., Wang M.Y., Uribe J.S. (2014). Introduction: Adult spinal deformity: Pathophysiology and corrective measures. Neurosurg. Focus.

[7] De la Garza Ramos R., Hamad M.K., Ryvlin J., Krol O., Passias P.G., Fourman M.S., Shin J.H., Yanamadala V., Gelfand Y., Murthy S. (2022). An Artificial Neural Network Model for the Prediction of Perioperative Blood Transfusion in Adult Spinal Deformity Surgery. J. Clin. Med..

[8] Asada T., Miura K., Koda M., Kadone H., Funayama T., Takahashi H., Noguchi H., Shibao Y., Sato K., Eto F. (2022). Can Proximal Junctional Kyphosis after Surgery for Adult Spinal Deformity Be Predicted by Preoperative Dynamic Sagittal Alignment Change with 3D Gait Analysis? A Case-Control Study. J. Clin. Med..

[9] Funao H., Yamanouchi K., Fujita N., Kado Y., Kato S., Otomo N., Isogai N., Sasao Y., Ebata S., Kitagawa Y. (2022). Comparative Study of S2-Alar-Iliac Screw Trajectories between Males and Females Using Three-Dimensional Computed Tomography Analysis: The True Lateral Angulation of the S2-Alar-Iliac Screw in the Axial Plane. J. Clin. Med..

[10] Endo H., Murakami H., Yamabe D., Chiba Y., Oikawa R., Yan H., Doita M. (2022). Comparison of Hybrid Posterior Fixation and Conventional Open Posterior Fixation Combined with Multilevel Lateral Lumbar Interbody Fusion for Adult Spinal Deformity. J. Clin. Med..

[11] Park S.C., Son S.W., Yang J.H., Chang D.-G., Suh S.W., Nam Y., Kim H.J. (2022). Novel Surgical Technique for Adolescent Idiopathic Scoliosis: Minimally Invasive Scoliosis Surgery. J. Clin. Med..

